# Within-season movements of Alpine songbird distributions are driven by fine-scale environmental characteristics

**DOI:** 10.1038/s41598-020-62661-0

**Published:** 2020-04-01

**Authors:** Francesco Ceresa, Mattia Brambilla, Juan S. Monrós, Franco Rizzolli, Petra Kranebitter

**Affiliations:** 1 Museum of Nature South Tyrol, Bolzano, Italy; 2 Museo delle Scienze, Sezione Zoologia dei Vertebrati, Trento, Italy; 3 Fondazione Lombardia per l’Ambiente, Settore Biodiversità e Aree protette, Seveso, MB Italy; 4 Institute Cavanilles of Biodiversity and Evolutionary Biology, University of Valencia, Paterna, Valencia, Spain

**Keywords:** Behavioural ecology, Ecological modelling, Animal behaviour

## Abstract

Information about distribution and habitat use of organisms is crucial for conservation. Bird distribution within the breeding season has been usually considered static, but this assumption has been questioned. Within-season movements may allow birds to track changes in habitat quality or to adjust site choice between subsequent breeding attempts. Such movements are especially likely in temperate mountains, given the substantial environmental heterogeneity and changes occurring during bird breeding season. We investigated the within-season movements of breeding songbirds in the European Alps in spring-summer 2018, using repeated point counts and dynamic occupancy models. For all the four species for which we obtained sufficient data, changes in occupancy during the season strongly indicated the occurrence of within-season movements. Species occupancy changed during the season according to fine-scale vegetation/land-cover types, while microclimate (mean temperature) affected initial occupancy in two species. The overall occupancy rate increased throughout the season, suggesting the settlement of new individuals coming from outside the area. A static distribution cannot be assumed during the breeding season for songbirds breeding in temperate mountains. This needs to be considered when planning monitoring and conservation of Alpine birds, as within-season movements may affect the proportion of population/distribution interested by monitoring or conservation programs.

## Introduction

Detailed information about the distribution and habitat use of organisms is essential for their conservation. Species distribution models are widely used to relate environmental and climatic variables to species occurrences, and the resulting relationships are used to predict species distributions in space and time^1^. This can provide useful information to assess the potential impact of environmental changes, identify priority areas for conservation, define ecological networks and design monitoring schemes^1,2^.

Distribution models have been largely used to investigate the distribution of bird species at different scales. In spite of the generally high mobility of birds, studies have generally assumed a static distribution during the breeding season^1^. However, several studies now indicate that within-breeding season movements (hereafter ‘within-season movements’) may be common, at least in multi-brooded species breeding in seasonal environments^3–10^. These movements probably represent displacements to higher quality breeding sites, occurring from one to the subsequent brood, or after a reproduction failure^3,11^. Habitat quality may change throughout the season^12^, as well as the cues available to birds to select a suitable breeding site^13^; in both cases, moving to more suitable areas would be an adaptive response. Within-season movements have been assessed in a broad variety of species with different reproductive behaviour and across many different scales (i.e. within study areas ranging from c. 1 up to c. 5000 km^2^ ^5,7,8,14,15,23^, or even across the entire breeding range of a species^16^). This is supported by some mark-resight and radio-tracking studies, which have reported within-season dispersal distances up to 17 km in the grey wagtail *Motacilla cinerea*^11^, and up to more than 40 km in the ortolan bunting *Emberiza hortulana*^17^. While mark-resight studies allow tracking single individuals, their application to large populations and over large areas would require a huge field effort. Most of the available information about within-season movements therefore has been obtained from distribution models based on surveys of unmarked birds. These distribution models allow detecting apparent movements based on distribution changes throughout the breeding season^3,14^. For species undertaking within-season movements, a poor knowledge of such dynamics can lead to biased research and monitoring results and, consequently, to inaccurate information available for conservation^1,6^. Biased estimates of site use, or inadequate study design for data collection under research or monitoring frameworks, can result from the assumption of static habitat use, if the breeding distribution of a bird species is actually dynamic^6,18,19^.

Within-season movements are especially likely in mountain areas in seasonal environments. In these areas, the elevational gradient and the complex orography, coupled with seasonal progression, result in environmental characteristics varying across short distances and within limited timeframes^20–24^. Several species in mountains perform altitudinal migratory movements^25^, but changes in spatial patterns of occurrence may occur also within the breeding season^3,4,8^. Within-season movements indeed may allow birds to track the within-season changes in habitat quality and thermal niche, or to adjust breeding site choice made by naive individuals^8,14^. In highly heterogeneous landscapes, species may persist in local ‘microrefugia’, in spite of changes occurring at larger scale^26,27^. Therefore, investigating within-season movements may also increase our understanding of the responses of mountain bird populations to environmental and climatic changes^28,29^. Whereas some studies provided evidence for within-season movements in mountain forests^8^ and middle-elevation open landscapes^3,4,30^, we lack information about these dynamics in high-elevation areas (treeline habitat, alpine grasslands and rocky uplands). More generally, fine-scale ecological studies about high-elevation bird species are scarce^29^. Considering also the pressures they are exposed to (because of climate change, land use changes and human disturbance^28,29,31^), more detailed information is urgently needed to inform monitoring and conservation and the related management policies.

**Figure 1:**
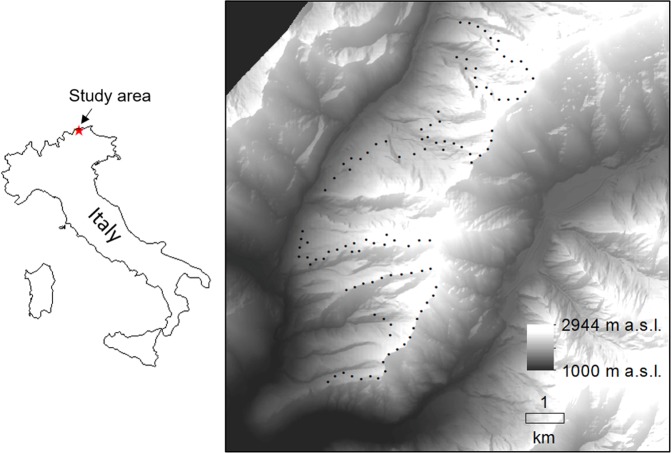
Location of the study area (central-eastern Alps, Italy) and distribution of the bird sampling points (N = 109).

In this study, we investigated the within-season movements of songbirds breeding in the European Alps. We focused on the breeding season (leaving out the post-breeding phase to avoid potential effects due to altitudinal migratory movements), and investigated changes in occupancy by means of repeated point counts (3 sampling sessions across 109 points) and dynamic occupancy models^32^. These models enable the estimation of initial occupancy (ψ) and the subsequent site colonization (γ) and extinction probability (ε), while accounting for imperfect detection (p). We hypothesized that occupancy, colonization (hereafter ‘settlement’) and local extinction (hereafter ‘vacancy’) could be influenced by land cover characteristics (structural vegetation types), by both land cover and microclimate (local mean temperature), or that they could be constant across the study area during the breeding season, and we built our model sets based on these alternative hypotheses (see Table 1). In order to test also the hypothesis of static distribution (i.e., no within-season movements), we also ran non-dynamic occupancy models, which do not estimate settlement and vacancy^18^. The study area (Fig. 1; elevation 1300–2700 m a.s.l.) ranged from valleys up to the highest local peaks, thus covering a broad and complete elevation gradient. Given this wide elevation range, the complex orography and the consequent high environmental heterogeneity in the study area, we expected to observe a dynamic rather than a static bird distribution throughout the breeding season. Within such a heterogeneous area, we also expected that land cover characteristics would affect the distribution dynamics, according to species-specific habitat requirements: higher settlement and lower vacancy probability could be expected at sites with favourable habitat characteristics, such as high cover of the preferred vegetation types (e.g. tree cover for forest species, grassland cover for open habitat dwellers).

**Table 1:** Occupancy models fitted to investigate the distribution dynamic of mountain-dwelling songbirds throughout the breeding season.

Model
ψ(.), p(.)
ψ(.), p(session + autocov)
ψ(lc), p(session + autocov)
ψ(lc + t), p(session + autocov)
ψ(.), γ(.), ε(.), p(.)
ψ(.), γ(.), ε(.), p(session + autocov)
ψ(lc), γ(.), ε(.), p(session + autocov)
ψ(lc + t), γ(.), ε(.), p(session + autocov)
ψ(lc), γ(lc), ε(lc), p(session + autocov)
ψ(lc + t), γ(lc), ε(lc), p(session + autocov)
ψ(lc), γ(lc + t), ε(lc + t), p(session + autocov)
ψ(lc + t), γ(lc + t), ε(lc + t), p(session + autocov)

Dynamic models describe initial occupancy (ψ), settlement (γ), vacancy (ε) and detection probability (p), while static models describe a time-constant occupancy accounting for detection probability. We compared models based on constant parameters (.) and on the influence of land cover characteristics (lc) and fine-scale temperatures (t), while for modelling detection we used the sampling session (session) and a temporal autocovariate (autocov).

We present results for four species: water pipit *Anthus spinoletta*, dunnock *Prunella modularis*, European robin *Erithacus rubecula* (hereafter robin) and coal tit *Periparus ater*. For other songbird species, models did not converge and/or they showed a severe lack of fit, probably because of the more reduced occurrence rate of most species in the study area. The four aforementioned species show different ecology and habitat associations. Water pipit is a mountain specialist and ground-nesting bird, breeding on alpine pastures and high-altitude meadows^33^. In the Alps, dunnock typically breeds in open montane forests and scrublands, usually avoiding dense and continuous woodlands, and is more common between 1400 and 2100 m a.s.l.^34^. Robin breeds in a variety of wooded habitats, preferring zones with a multi-layered undergrowth, with shrubs of different height; in the Alps, it nests from valley bottoms up to 2000–2100 m a.s.l.^35^. Coal tit is a forest-dwelling and cavity-nesting bird, which mainly breeds in coniferous woodlands. Per breeding season, the water pipit rears one to two broods^33^, while the other species are double-brooded and can occasionally rear a third brood^34–38^.

## Results

The models obtained through an information-theoretic approach showed that dynamic occupancy models clearly performed better than static models in all four species (Table 2). In fact, only models describing a dynamic occupancy were substantially supported (ΔAIC < 2)^39^. Furthermore, comparing the best dynamic and the best static model for each species, the dynamic model was always significantly more supported according to the likelihood ratio test (water pipit: *χ*^2^ = 75.60, df = 6, p < 0.001; dunnock: *χ*^2^ = 14.69, df = 6, p = 0.023; robin: *χ*^2^ = 39.05, df = 7, p < 0.001; coal tit: *χ*^2^ = 41.28, df = 6, p < 0.001). We did not find evidence for significant lack of fit in the top-ranked models (those with ΔAIC < 2) according to a goodness-of-fit test based on the Pearson’s chi-square statistics^40^ (p > 0.05 in all cases). We also found no evidence for influence of spatial autocorrelation on occupancy (Moran’s I range: −0.109–0.014; p > 0.05 in all cases).

**Table 2:** AIC-ranked models describing initial occupancy (ψ), settlement (γ), vacancy (ε) and detection probability (p) during the breeding season of water pipit (WP), dunnock (DU), robin (RO) and coal tit (CT).

Species	Model	AIC	ΔAIC	K
WP	ψ(grs + bush), γ(grs + bush), ε(grs + bush), p(session + autocov)	566.67	0.00	13
	ψ(grs + bush), γ(grs + bush + t), ε(grs + bush + t), p(session + autocov)	566.79	0.12	15
	ψ(grs + bush + t), γ(grs + bush), ε(grs + bush), p(session + autocov)	566.84	0.17	14
	ψ(grs + bush + t), γ(grs + bush + t), ε(grs + bush + t), p(session + autocov)	567.16	0.49	16
	*ψ(grs* + *bush), p(session* + *autocov)*	*630.2*7	*63.60*	*7*
DU	ψ(trs + bush + t), γ(trs + bush), ε(trs + bush), p(session + autocov)	535.08	0.00	14
	*ψ(trs* + *bush* + *t), p(session* + *autocov)*	*537.77*	*2.69*	8
RO	ψ(trs + bush + t), γ(trs + bush), ε(trs + bush), p(session + autocov)	358.90	0.00	14
	ψ(trs + bush), γ(trs + bush), ε(trs + bush), p(session + autocov)	360.07	1.17	13
	*ψ(trs* + *bush), p(session* + *autocov)*	*383.95*	*25.05*	*7*
CT	ψ(grs + bush), γ(grs + bush), ε(grs + bush), p(session + autocov)	457.23	0.00	13
	ψ(grs + bush + t), γ(grs + bush), ε(grs + bush), p(session + autocov)	458.30	1.07	14
	*ψ(grs* + *bush), p(session* + *autocov)*	*486.51*	*29.28*	*7*

The best static occupancy model (describing only ψ and p) for each species is reported in italics. Covariates included in the models: mean temperatures (t), extension of grassland (grs), bushes (bush) and trees (trs), sampling session (session), temporal autocovariate (autocov).

**Figure 2:**
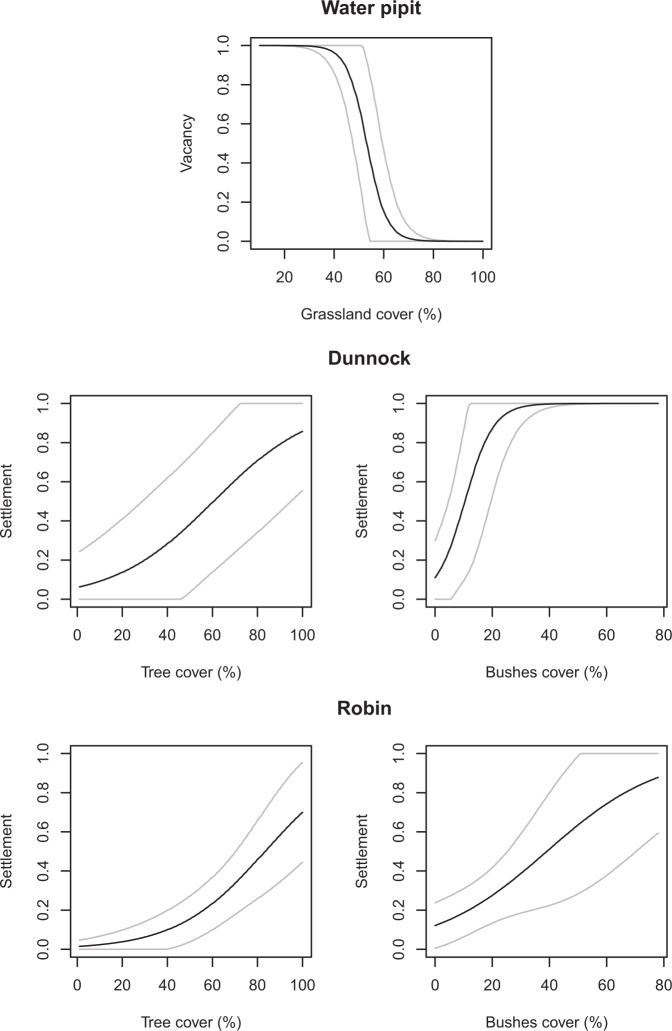
Effects of land cover characteristics on the probability of settlement (γ) or vacancy (ε) of mountain-dwelling songbirds during the 2018 breeding season, based on dynamic occupancy models. Only significant effects are reported. Gray lines represent 95% confidence intervals.

**Figure 3:**
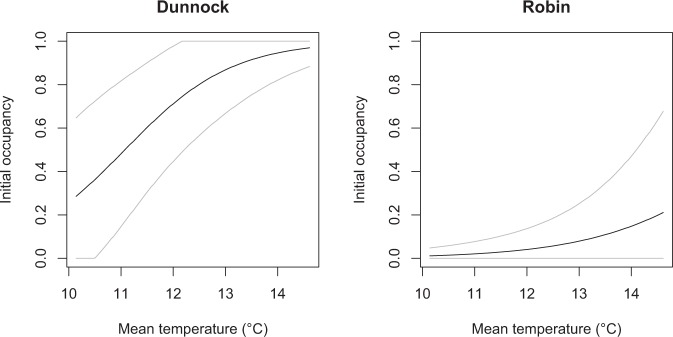
Effects of local temperatures on the initial occupancy probability (early in the breeding season) of Dunnock and Robin. Only significant effects are reported. Gray lines represent 95% confidence intervals.

**Figure 4:**
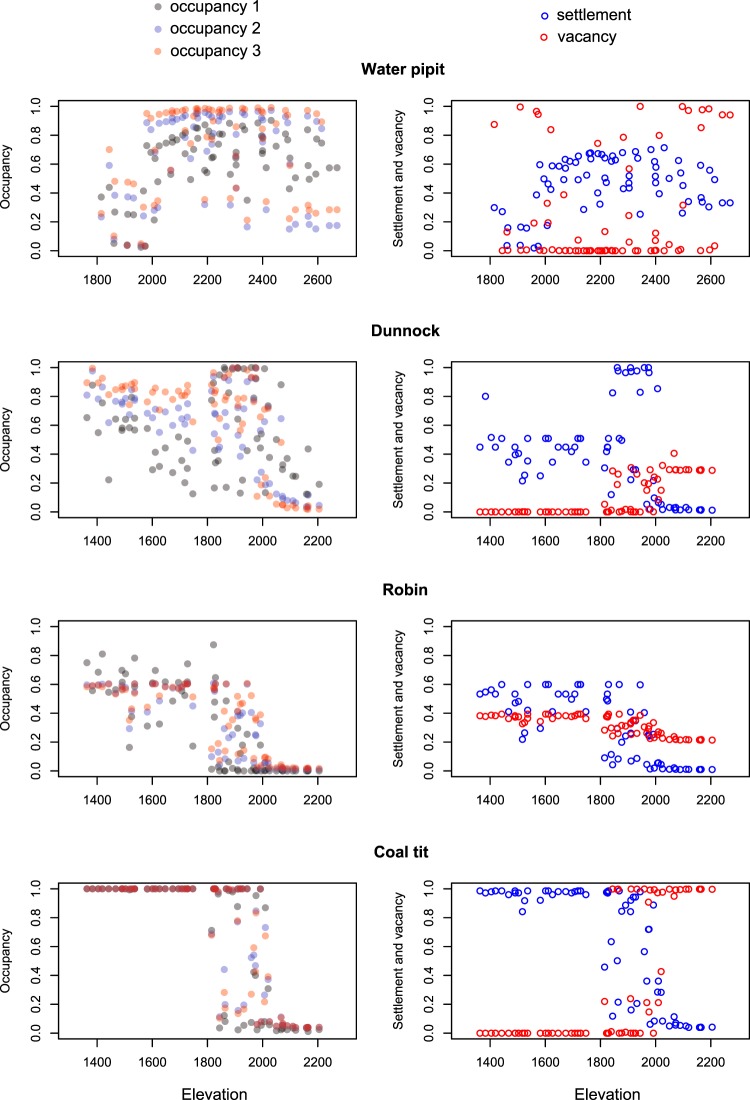
Occupancy, settlement and vacancy probability of four songbird species along the elevation gradient during the 2018 breeding season, based on the most parameterized top-ranked dynamic occupancy model for each species (see Table 2). Initial occupancy ψ (occupancy 1), settlement (γ) and vacancy (ε) were directly estimated in the models, and were used to subsequently calculate the occupancy probability during the following sampling sessions (occupancy 2 and occupancy 3).

In all top-ranked models, initial occupancy, settlement and vacancy varied according to land cover characteristics and, in some cases, also to mean temperature. To evaluate predictor effects (Table 3), for each species we considered the model with the highest number of parameters (K), among those with ΔAIC < 2. We found a significant effect (95% confidence intervals not encompassing zero) of land cover on settlement or vacancy for water pipit, robin and, less clearly (based on 90% confidence intervals), for dunnock (Table 3). More specifically, for water pipits the probability of vacancy declined with greater grassland cover (Fig. 2), hence promoting the occurrence of this species through time. For robins and dunnocks, settlement probability increased with greater cover of tree canopy and of bushes (Fig. 2). In both these species, settlement probability was close to 0.1 with 1% tree cover, and increased up to 0.7–0.8 with 100% tree cover (Fig. 2); with an 80% increase in bushes cover, settlement probability increased nine fold for dunnocks and seven fold for robins (Fig. 2). When included in the top-ranked models, the effect of mean temperature was not significant on the dynamic parameters, but it positively affected the initial occupancy of dunnock and, less clearly (based on 90% confidence intervals), of robin (Table 3). With a mean temperature increase from 10.1 to 14.6 °C, the initial occupancy probability increased three fold for dunnocks and five fold for robins (Fig. 3). In all species but robin, the mean settlement probability in an unoccupied point was higher than the probability of vacancy from an occupied point (mean ± SE: water pipit: γ = 0.46 ± 0.02, ε = 0.26 ± 0.05; dunnock: γ = 0.41 ± 0.04, ε = 0.10 ± 0.02; robin: γ = 0.30 ± 0.03, ε = 0.32 ± 0.07; coal tit: γ = 0.65 ± 0.04, ε = 0.35 ± 0.06). These dynamics resulted in a higher mean occupancy in the second and third sampling session compared to the initial occupancy, although such increase was negligible in the coal tit (Table 4). Given the effect of vegetation and temperature, these changes were not spatially uniform, and showed clear elevational patterns in the magnitude of variation in occurrence and in differences between settlement and vacancy probability (Fig. 4). Forest-dwelling species showed highly dynamic distributions in the transitional belt around the timberline (1800–2000 m a.s.l.), between the subalpine woodland and the high-altitude open areas. Their spatio-temporal patterns differed at lower elevation, with a prevailing high occupancy and temporal stability in the coal tit, colonization in the dunnock and a reduction of spatial variability in occupancy in the robin. Finally, the water pipit was more likely to vacate sites at its lower and upper elevational occurrence limits (Fig. 4).

**Table 3:** Parameter estimates (Est) and standard errors (SE) for the most parametrized top-ranked occupancy models describing initial occupancy (ψ), settlement (γ), vacancy (ε) and detection probability during the three sampling sessions (p_1_, p_2_ and p_3_) in four songbird species: water pipit (WP), dunnock (DU), robin (RO) and coal tit (CT).

Variables		Species							
		WP		DU		RO		CT	
		Est	SE	Est	SE	Est	SE	Est	SE
ψ	Intercept	0.60	0.32^†^	0.62	0.64	−3.36	1.29^*^	4.03	4.20
	Grassland	0.87	0.40^*^					−5.42	4.06
	Bushes	−0.54	0.42	2.86	1.42^*^	0.14	1.90	−2.60	1.74
	Trees			0.22	0.39	3.55	1.41^*^		
	Temperature	−0.44	0.34	1.00	0.46^*^	0.72	0.44^†^	−0.69	0.87
γ	Intercept	−0.22	0.43	−0.34	0.73	−1.54	0.43^*^	1.19	3.12
	Grassland	0.65	0.50					−2.74	2.51
	Bushes	−0.56	0.45	3.51	1.85^†^	0.89	0.39^*^	−0.66	1.12
	Trees			1.83	0.98^†^	2.05	0.63^*^		
	Temperature	−0.15	0.37						
ε	Intercept	−4.40	2.20^*^	−7.36	9.66	−0.80	1.43	−5.33	3.50
	Grassland	−6.33	2.69^*^					7.85	6.66
	Bushes	−2.98	1.85	−0.70	0.86	0.10	1.94	4.25	3.52
	Trees			−6.58	8.41	0.36	1.65		
	Temperature	2.27	1.45						
p_1_	Intercept	0.87	0.29^*^	−0.27	0.28	1.42	0.51^*^	0.34	0.27
p_2_	Intercept	1.35	0.30^*^	−0.29	0.26	0.06	0.72	0.35	0.23
p_3_	Intercept	−0.68	0.21^*^	−1.60	0.24^*^	−0.14	0.43	0.97	0.27^*^
	Autocovariate	0.94	0.27^*^	1.16	0.29^*^	1.32	0.53^*^	1.70	0.29^*^

^*^95% confidence interval does not include 0; ^†^90% confidence interval does not include 0.

**Table 4:** Mean occupancy probability ψ (±SE) of water pipit (WP), dunnock (DU), robin (RO) and coal tit (CT), estimated for three sampling periods during the breeding season of year 2018.

Species	30 May – 6 June	19–23 June	13–18 July
WP	0.62 ± 0.03	0.64 ± 0.04	0.69 ± 0.04
DU	0.54 ± 0.04	0.59 ± 0.04	0.66 ± 0.04
RO	0.27 ± 0.04	0.33 ± 0.03	0.36 ± 0.03
CT	0.66 ± 0.05	0.68 ± 0.05	0.67 ± 0.05

## Discussion

For all the four songbird species for which we obtained reliable results, we found evidence for within-season movements in our study area. All four species are abundant or very abundant in the study area and, more generally, in the Alps; this makes unlikely that, in the core of the breeding season, the occurrence of unpaired or prospecting males could bias our occupancy estimates. The evidence for within-season movements is consistent with previous studies, undertaken at lower elevations, with other bird species, mainly in forests and farmlands^3–6,8,14,41^. Our results further suggest that within-season movements and/or dynamic distributions could be common among multi-brooded birds breeding in seasonal environments. Furthermore, we observed these dynamics in four species with clearly different ecological requirements, supporting this suggestion. Similarly to previous studies^3–6,8,14,41^, the evidence we obtained for within-season movements is indirect, as we did not mark single individuals. For this reason, besides within-season movements, in this section we will also evaluate and discuss all possible alternative explanations for the observed changes in occupancy.

The observed patterns could be the result of different and possibly combined processes. Individuals may have moved after nest failure^11,42^ and settled in other sites to attempt a substitution clutch, trying to select the new breeding site in more suitable areas. Cues about habitat suitability may be insufficient at the beginning of the season^14,43^, leading some birds to settle in suboptimal areas, especially in the case of unexperienced individuals. Also successfully breeding birds may choose different breeding sites for the subsequent clutches, in order to adjust for possible environmental changes occurred with the progress of the season, such as temperature changes (see iButtons recordings summarized in the Supplementary Information), vegetation development^21^ and changes in prey abundance^24,44^.

The overall increase of the occupancy probability throughout the season suggests that also the arrival of individuals from outside the study area could contribute to this dynamic pattern of site occupancy. For the species we investigated, late arrivals from wintering areas are unlikely, because the migratory periods of these songbirds largely precede our study period. The breeding populations of coal tit are likely to be largely resident within the study area. Alternatively, this pattern could be explained by late settlements of individuals looking for a territory/mate, possibly as part of within-season dynamics on a larger scale than the extent actually covered by our study (as described in some previous studies^5,17^).

The overall environmental conditions may also have improved throughout the season, allowing the settlement of more breeding pairs^41^. Robins and dunnocks selected warmer sites at the beginning of the season, suggesting that these areas provided environmental conditions that allow breeding early. This potentially implies several advantages, as breeding early can increase reproduction success^45,46^, can provide competitive advantages for early born individuals^47,48^ and results in a larger time window for subsequent reproduction attempts. Later in the season, settlement probability of both species was affected only by the availability of wooded and bushy areas (Tables 2 and 3), and the overall occupancy increased.

The highly variable occupancy patterns in the transitional belt between woodland and alpine grassland indicate that extent of suitable habitat available for each species varies within the season, and this in turn determines a complex scenario of settlement and abandonment throughout the season. Besides climate and topography, also cattle grazing, forestry and frequent snow slides strongly shaped the vegetation structure around the transitional belt connecting forest and alpine grassland. These factors created a mosaic landscape with alternating grasslands and wooded/bushy areas. Given the fragmented suitable habitat, this is probably a sub-optimal zone for water pipits^49^ and for species preferring continuous woodlands^50,51^, and may be occupied by less competitive individuals. Conversely, such a landscape is especially suitable for dunnocks, as reported in the literature^34,51^ and confirmed by high occupancy and settlement probability (Fig. 4).

Dunnocks and water pipits tended to vacate their occurrence sites at higher elevation, placed respectively between 2000 and 2200 and above 2500 m a.s.l. (Fig. 4). This is possibly due to an insufficient extent of suitable habitat at these altitudes: between 2000 and 2200 m grassland becomes dominant, while the cover of tree/bushes available for dunnocks is very low. Similarly, above 2500 m the cover of rocky areas definitely increases, while the cover of grassland available for water pipits is low. For the water pipit, the negative effect of grassland extension on vacancy probability supports this hypothesis (Table 3). Settlement occurred in poorly suitable areas by unexperienced or poorly competitive individuals could contribute to explaining these patterns. Summer storms (occurred within the study season in the study area) with snow at high elevation might also contribute to the observed vacancy of high-elevation sites. However, we did not find any significant effect of mean temperatures on the vacancy probability of these species (Table 3). In the case of water pipit, mean temperature is included in some top-ranked models, but with no significant effects (Table 3).

Overall, we found non-significant or null effects of fine-scale temperature on settlement and vacancy in our study area. This suggests that the effect of variation in local temperatures on primary factors for breeding birds (e.g. prey availability, vegetation development) was not strong enough to determine changes in occupancy throughout the breeding season. We could not evaluate the potential effects of other climate/weather variables, such as precipitations, which can also affect bird reproduction^52^. Frey *et al*.^8^ on the other hand found an overall high influence of fine-scale temperature metrics on within-season movements in a set of 15 forest bird species. Anyway, in Frey *et al*.^8^ the influence of microclimate varied between the two study years and across species. The patterns we observed are based on a single breeding season, and could change according to annual conditions. Future studies based on more sample years may allow to better understand the factors affecting the within-season movements of breeding birds, and especially the role of local climate, which shows high inter-annual variations^8^. Broadening the time span of studies would allow to obtain more reliable results for a higher number of species, because increasing the number of sampling occasions improves the accuracy and precision of parameter estimation in occupancy models^32^. Including also pre- and post-breeding periods would allow an explicit assessment of altitudinal migratory movements too. Therefore, future broader, multi-year studies are highly welcome for dynamic environments like temperate mountains.

As noted in other studies using dynamic occupancy models^8,14^, settlement and vacancy probability may be affected by movements of individuals within breeding territories that do not entirely fall into the sampling plots. Even controlling for imperfect detectability, this may result in apparent site settlements and abandonments while individuals are simply moving into and outside the sampling plots, without leaving their territories (‘temporary emigration’). We partly addressed this issue by using sampling plots with an area of 3.14 ha, which is similar or larger than the territory size of our study species (approx. 0.5–3 ha^37,53–56^). This increased the probability that breeding territories were entirely included within the sampling plots. In any case, temporary emigration is likely to bias estimates only if it is not random^57^. Also sampling migratory individuals could result in dynamic distributions that are not determined by changes of breeding sites, but by arrivals and departures of transient birds. However, while we could have contacted some delayed transient individuals of long-distance migratory species (e.g., garden warbler *Sylvia borin*, Western Bonelli’s warbler *Phylloscopus bonelli*), our study period did not overlap with the migration time of the four modelled species, which are short/medium distance or altitudinal migratory birds^58^ or probably mostly resident in the case of the coal tit. Even if altitudinal migration is in fact common in many mountain bird species, and alpine habitats are intensively exploited by several species in the post-breeding and/or fall migration periods^59^, by focusing on the strict breeding period of our study species we feel confident that the patterns we found are related to within-season movements. Therefore, while we cannot completely exclude that we sampled some exceptionally late migratory individuals, it is extremely unlikely that this could affect our results.

Our findings suggest that a static distribution cannot be safely assumed for Alpine songbirds, which occupy environments subject to important within-season variations, and include many multi-brooded species. Ignoring the dynamic occupancy patterns shown by those species may lead to biases in habitat use estimates^18,19^, possibly providing also biased information for bird conservation. Repeated sampling, at least early and late during the breeding season, is therefore needed to obtain more reliable information about bird distribution and habitat use. In our case, surveying birds only early in the season would have led to a general underestimation of bird occupancy and biased results about habitat use (e.g., a large underestimation of dunnock occupancy in the forested part of the study area). Through a dynamic occupancy approach, we obtained reliable results for only four common songbirds. However, obtaining such detailed information also for less abundant and more endangered species would be particularly useful for conservation purposes. Besides more sampling occasions, also higher detection probabilities would allow better estimations^32^. The latter could be achieved through more targeted sampling designs, for example by focusing sampling effort on specific habitats, or by adopting species-specific sampling methods such as playback use. In addition, as proposed by Gomez *et al*.^60^ for single-season abundance models, using a multi-species approach to calculate detectability may allow better estimates also for rare species.

Our results suggest the occurrence of distribution dynamics at a larger scale than our study area; future investigation considering wider Alpine areas (e.g. large protected areas) could increase our knowledge about these dynamics, and ideally provide temporally explicit distribution maps that could be useful for planning bird monitoring and for habitat management (e.g. temporary restrictions of activities potentially harmful for breeding birds).

The occupancy patterns observed in our study area indicate that settlement of the study species occurred not only at the beginning of the breeding season, but also afterwards. Breeding territory establishment is a crucial and sensitive period, and human disturbance can affect the birds’ decision to settle^61^. Therefore, the increasing popularity of a variety of outdoors activities in mountain areas could affect birds’ distribution dynamics, besides other possible negative effects such as increasing stress levels^62^ and avoidance of potentially suitable habitat^63^. Many undisturbed sites at the beginning of the season are likely to become more disturbed later, given that in the Alps highly popular activities like hiking and biking are more practised in summer than in spring. This could result in a reduced possibility of tracking favourable habitat conditions, with more limited site choice for second or replacement clutches. Our study area is poorly frequented by outdoor recreationists, and studies comparing areas with different levels of disturbance are needed to assess the impact of outdoor activities on this and other aspects of the breeding ecology of mountain songbirds.

In conclusion, our study displayed dynamic breeding distributions for four Alpine songbirds with divergent habitat preferences. This challenges the traditional idea of a static distribution during the breeding season, especially in highly dynamic environments like mountains in temperate regions. Further multi-year studies covering larger areas could shed light on the overall importance of dynamic occurrence patterns in breeding birds.

## Methods

### Fieldwork

Data collection took place in the central-eastern Italian Alps (Wipptal, South Tyrol), immediately south of the main Alpine divide, within a 3400 ha-wide area (46.96°N, 11.50°E). This area spans from approximately 1300 m a.s.l. (just above the bottom valley) to more than 2700 m a.s.l. at the highest peaks. At lower elevation, mountainsides are mostly covered by woodlands, dominated by spruce *Picea abies* and European larch *Larix decidua*. Above the timberline (approximately 2000 m a.s.l., but highly variable), wide areas are covered by bushes (mainly *Rhododendron* spp.) and scattered larches, while alpine grasslands, rocks and scree slopes characterize the upper elevation belt. The local climate is continental, with strong temperature variation between winter and summer and between day and night, and with precipitation concentrated mostly in the summer months^64,65^.

We surveyed birds by means of point counts (N = 109, Fig. 1), carried out during the breeding season of most local breeding species (May – July), during year 2018. Surveys were performed by expert field ornithologists with large experience in acoustic and visual identification of local avifauna, and in use of the point counts technique. The rugged topography, and the consequent low accessibility of some areas, made a random selection of the sampling points unfeasible. Therefore, we carried out the point counts along accessible transects, often along footpaths; we chose the location of each point based on a minimum distance of 200 m to the previous one, in order to avoid potential replicated counts of the same individuals^49^.

At each sampling point, the surveyor recorded all birds observed or heard during 10 min within a 100 m radius. Each 10-min count was divided into three 3-min 20-s sub-counts, starting a new and distinct sampling period at the beginning of each sub-count^14^. The division into sub-counts was performed because it allows the estimation of detection probability within the framework of dynamic occupancy models, and also meets the assumption of population closure^32^ (see details in “Statistical analysis”). All points were visited three times, corresponding to three distinct survey sessions: 30 May – 6 June, 19–23 June and 13–18 July. The timing of the first session was chosen to match the first brood period of most species, in an area characterized by a severe winter/early spring climate and long-lasting snow cover (large parts of the study area were still covered by snow in early May). This survey timing also allowed excluding the main migration periods (considering also altitudinal migration), a key point to avoid the influence of migratory individuals on the investigated dynamics. Counts were carried out in the morning, between 5:30 and 11:30, and avoiding poor weather conditions (i.e., rain or strong wind).

Vegetation and other land cover characteristics within a 100 m radius were recorded at each sampling point by the surveyor. In the field, with the aid of a 1:2000 map with the 100 m radius superimposed to the aerial orthophotograph, the observer estimated the percentage cover of selected variables (a map example is provided in the Supplementary Information; orthophotographs were made available by the Autonomous Province of Bolzano). These variables included the percentage cover of tree canopy (i.e. vegetation higher than 2 m), bushes (woody vegetation lower than 2 m, mostly represented by *Rhododendron* shrublands), grassland (areas with no canopy and covered by grassland vegetation) and rocks/scree (emerging bedrock or scree-covered patches). The percentage cover of each variable was thus visually estimated in the field to the nearest 5%. Variables covering less than 5% of the plot surface (as defined by the 100 m radius) were assigned a 1% cover value. In the woodland, where the visibility within 100 m can be reduced, the orthophotographs allowed detecting areas not covered by the tree canopy (clearings, rocky and bushy areas). Orthophotographs were also helpful in the transitional belt, as the difference between tree canopy and *Rhododendron* shrubland is clearly visible.

In addition, in order to obtain also microclimatic information, at each sampling point we placed an iButton data logger (models DS1921G and DS1922L, Maxim Integrated, San Jose, CA), which recorded temperatures hourly during the entire study period. Loggers were placed at the ground level and were protected from the direct solar radiation by means of a white plastic panel. Further details about loggers’ placement and use, as well as the temperature trends recorded at each point during the study period, are provided in the Supplementary Information.

### Statistical analysis

Dynamic occupancy models were fitted using program PRESENCE ver. 2.12.20^66^. These models use detection histories from multiple surveys (in our case, the sub-counts) over multiple ‘seasons’ (i.e. the three sampling sessions in our study) to estimate: the initial site occupancy, site colonization, site extinction and detection probability^32^. Therefore, this approach allows a separate modelling of the ecological processes (occupancy and its dynamics) and of the observation process (detection probability). Accounting for imperfect detection is of critical importance to obtain reliable estimations of species occurrence probability and of their distribution dynamics^18,32^; in dynamic occupancy models, detectability can be estimated thanks to the multiple surveys within each season (see MacKenzie *et al*.^32^ for the statistical details). Site colonization and extinction are calculated between seasons (the sampling sessions in our case), while population closure (no occupancy changes) is assumed within each season (i.e. session)^32^; this is a reasonable assumption in our study, given that the sub-counts were consecutive within a 10-min period. Considering the three seasons and the three sub-counts, we overall obtained nine sampling occasions per point. Consequently, our detection histories consisted in vectors of nine detection (1) or non-detection (0) records. We based our study on occupancy rather than on abundance in order to obtain more robust estimates (abundance estimation is likely to be more strongly affected by sampling biases than occupancy); in addition, we did not expect large variation in bird abundance within our small sampling units, which can include a very few breeding territories of the study species.

We obtained reliable results for the four species treated in the Results, while for the other songbird species the models often failed to converge and/or showed a severe lack of fit. This happened for all species detected at less than 35 points, indicating that the available data were probably too scarce. Species detected at less than 20 points (25 species out of the total 41 detected songbird species) were not considered in the analysis. The list of all bird species detected at the sampling points is available as Supplementary Information.

To avoid the inflation of uninformative zeroes, for each species we excluded from the analysis the clearly unsuitable sampling points, based on the ecological requirements of the study species (see the references in the Introduction) and the percentage cover of our land cover variables within 100 m radius. For species that use trees or shrubs as nesting sites (see del Hoyo *et al*.^67^), we excluded all sampling points with tree canopy cover = 0, i.e., we used only those points placed in the forest or in the transitional belt (N points = 68). Of the open habitat species breeding in the study area, we obtained reliable results about the commonest one, the water pipit. We recorded this species at points with a 32% maximum cover of trees; following Chamberlain *et al*.^68^, we therefore excluded from the analysis all points with tree canopy cover >40% (i.e., we used 70 points covered by open and semi-open habitat). Following the same approach, for the wheatear *Oenanthe oenanthe* we excluded all points with tree canopy cover >10% (canopy cover in occurrence points always <5%), but for this species we did not obtain reliable results. We built our model set based on a series of alternative hypotheses of increasing complexity^32^ (listed in Table 1), including also non-dynamic occupancy models (describing the static distribution hypothesis). For each species, we included those land cover variables that we expected to be important, based on the species’ breeding ecology^14,19^. As microclimatic predictors, for the initial occupancy we used the mean temperature at each point during the entire first sampling session, while for settlement and vacancy we used the mean temperature calculated over the periods between the three sessions (i.e., 7–19 June and 24 June-12 July). We used mean temperature over these periods because it allows describing the general climatic conditions at a location^8,69,70^. We expected the consecutive sub-counts to be temporally autocorrelated, thus to model detection probability we added a temporal autocovariate, indicating if the species was detected during the previous sub-count or not, during the same 10-min count. We also expected detection probability to vary across sampling sessions, because of the changing bird activities and behaviours throughout the breeding season (e.g., varying song rates, incubation periods and nestling feeding). Therefore, we modelled detection using both sampling session and the temporal autocovariate. All continuous predictor variables were standardized (z-transformation) to improve the software performance^71^ and to allow the comparison of effect sizes. We did not include strongly correlated variables in the same model (r < |0.5| for all pairs of variables). Models were ranked according to their Akaike information criterion (AIC), and those showing the lower AIC values (with ΔAIC < 2) were considered to be substantially supported^39^. To test the hypothesis of static occupancy with the hypothesis of dynamic occupancy, for each species we also performed a likelihood ratio test with the best non-dynamic model (null hypothesis) and the best dynamic model. Initial occupancy, settlement and vacancy probability obtained from dynamic models were used to calculate the occupancy probability during the second and third sampling sessions^71^, in order to achieve a clear representation of the distribution changes.

For each species, we assessed the goodness-of-fit of the top-ranked models through the parametric bootstrapping approach implemented in program PRESENCE: a Pearson chi-square fit statistics (sum of the square errors) comparing observed and expected values was calculated using the original fitted model and 250 simulated data sets. P values < 0.05 indicate a significant lack of fit^40^. For the top-ranked models, we also tested for spatial autocorrelation in occupancy by calculating Moran’s I for model residuals^72^ using ArcGis 10.5 (ESRI, Redlands, CA).

## Supplementary information

Supplementary Information.

## Data Availability

The datasets generated during and/or analysed during the current study are available from the corresponding author on reasonable request.
