# Supplementary material for: Within-season movements of Alpine songbird distributions are driven by fine-scale environmental characteristics
Source: Sci Rep. 2020 Apr 1;10:5747. doi: 10.1038/s41598-020-62661-0 (PMC7113314; doi:10.1038/s41598-020-62661-0)

Within-season movements of Alpine songbird distributions are driven by fine-scale environmental characteristics

Francesco Ceresa[1*], Mattia Brambilla[2,3], Juan S. Monrós[4], Franco Rizzolli[1] & Petra Kranebitter[1]

[1]Museum of Nature South Tyrol, Bolzano, Italy

[2]Museo delle Scienze, Sezione Zoologia dei Vertebrati, Trento, Italy

[3]Fondazione Lombardia per l'Ambiente, Settore Biodiversità e Aree protette, Seveso, MB, Italy

[4]Institute Cavanilles of Biodiversity and Evolutionary Biology, University of Valencia, Paterna (Valencia), Spain

Supplementary Data

List of all bird species detected during the point counts.

Tetrastes bonasia
Lyrurus tetrix
Lagopus muta
Falco tinnunculus
Cuculus canorus
Apus apus
Tachymarptis melba
Dendrocopos major
Alauda arvensis
Delichon urbicum
Anthus spinoletta
Anthus trivialis
Troglodytes troglodytes
Prunella collaris
Prunella modularis
Erithacus rubecula
Phoenicurus ochruros
Saxicola rubetra
Oenanthe oenanthe
Turdus merula

Turdus philomelos
Turdus torquatus
Turdus viscivorus
Sylvia atricapilla
Sylvia borin
Sylvia curruca
Phylloscopus bonelli
Phylloscopus collybita
Regulus regulus
Cyanistes caeruleus
Periparus ater
Poecile montanus
Lophophanes cristatus
Sitta europaea
Certhia familiaris
Garrulus glandarius
Nucifraga caryocatactes
Pyrrhocorax graculus
Corvus corone
Corvus corax
Montifringilla nivalis
Fringilla coelebs
Serinus serinus
Chloris chloris
Spinus spinus
Linaria cannabina
Acanthis flammea
Loxia curvirostra
Pyrrhula pyrrhula

Supplementary Methods

Temperature loggers placement and use

We recorded air temperature at each census point by means of iButton data loggers (Maxim Integrated, San Jose, CA), models DS1921G (N = 99) and DS1922L (N = 10). We programed temperature to be recorded every 60 min. Loggers were placed in the study area between 24 and 29 May and were recovered between 13 and 18 July 2018. Each data logger was mounted on a plastic fob (model DS9093A, Maxim Integrated, San Jose, CA), which was fixed on the ground by a stake. Data loggers were protected from direct solar radiation by means of a white plastic panel (approx. 20x20 cm). In a few cases, these sun shields were damaged or removed, possibly by people or by

cattle, and one iButton was no longer found. Data visualization confirmed that loggers with damaged sun shields had been exposed to direct solar radiation; these data were excluded from the following analysis.

Supplementary Figure 1: Daily mean temperatures at sampling points (N = 109; grey lines) during the study period, obtained from hourly temperatures recorded by iButtons data loggers. The red line represent the daily mean temperature, averaged across all sampling sites.

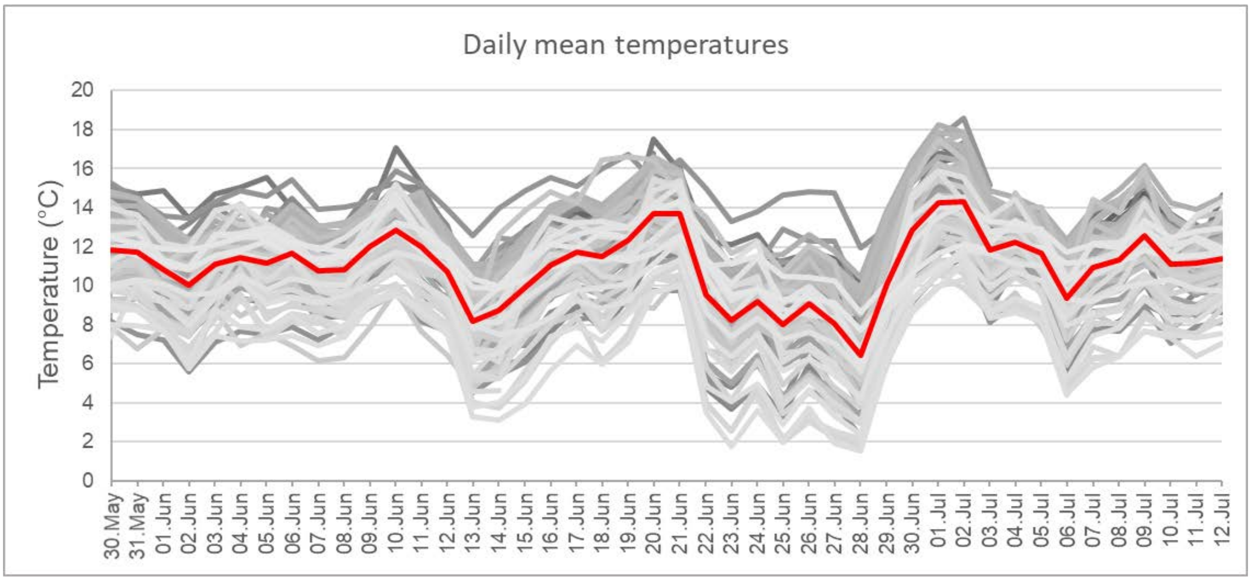

Supplementary Figure 2: Example of map used in the field by the surveyors at each sampling point (red dots), as an aid to visually estimate the extension of land cover categories within 100 m radius (black circles). The ortophotographs have been made available by the Autonomous Province of Bolzano, Ripartizione 28 (http://www.provincia.bz.it/natura-ambiente/natura-territorio/pianificazione-paesaggistica-territoriale.asp).

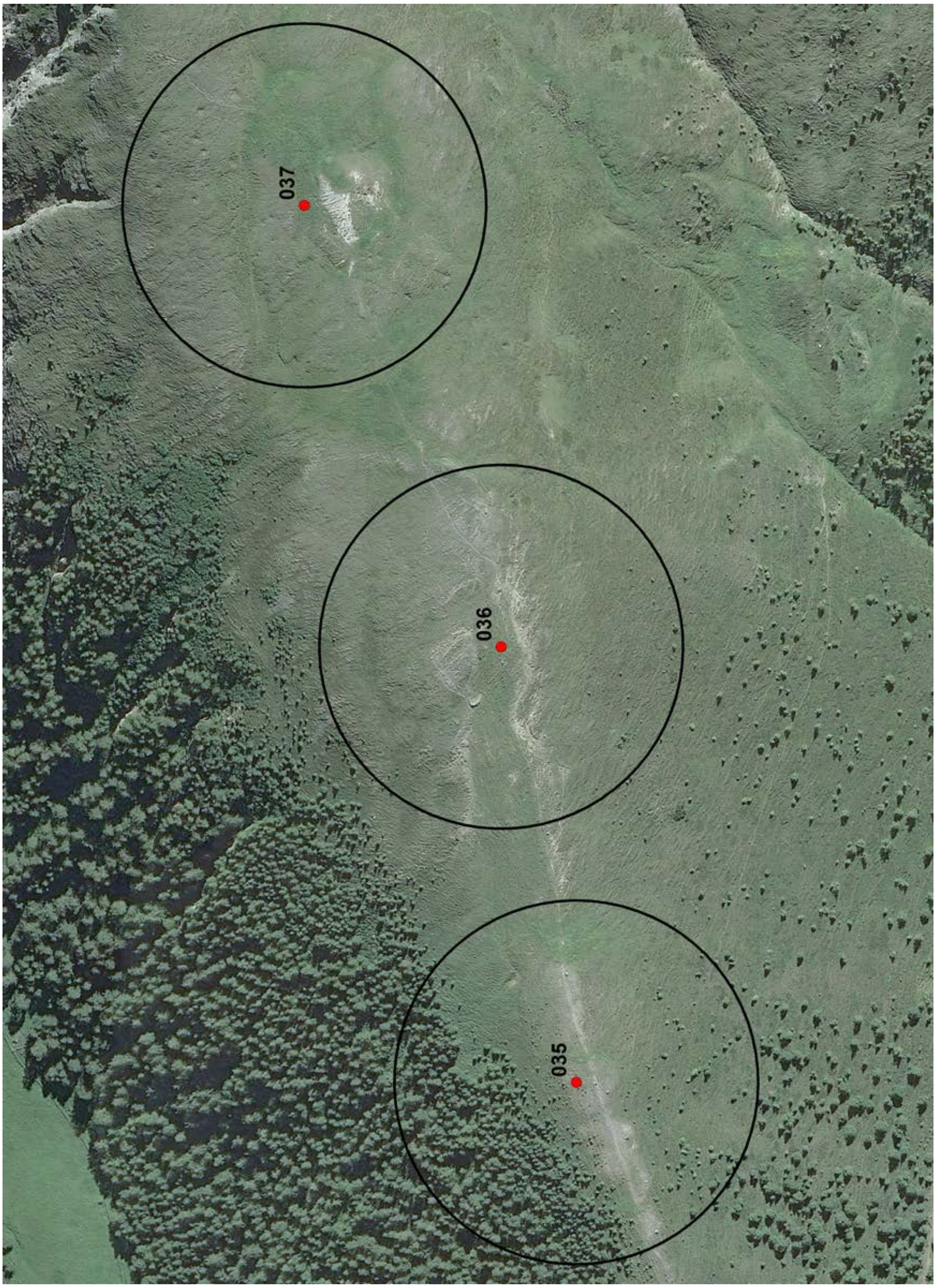

Supplement: Supplementary file 1 — Supplementary Information. [file 41598_2020_62661_MOESM1_ESM.pdf]
